# Supplementary material for: Detection of Equus Caballus Papillomavirus Type-2 in Asymptomatic Italian Horses
Source: Viruses. 2022 Jul 31;14(8):1696. doi: 10.3390/v14081696 (PMC9412442; doi:10.3390/v14081696)

Figure S3 : Raw reads aligned to either the reference EcPV2 genome or on the reconstructed sequences and visualized through the Integrative Genome Viewer (IGV)

a) ID2396 1-1 raw reads aligned to the EcPV2 reference LC612601.1

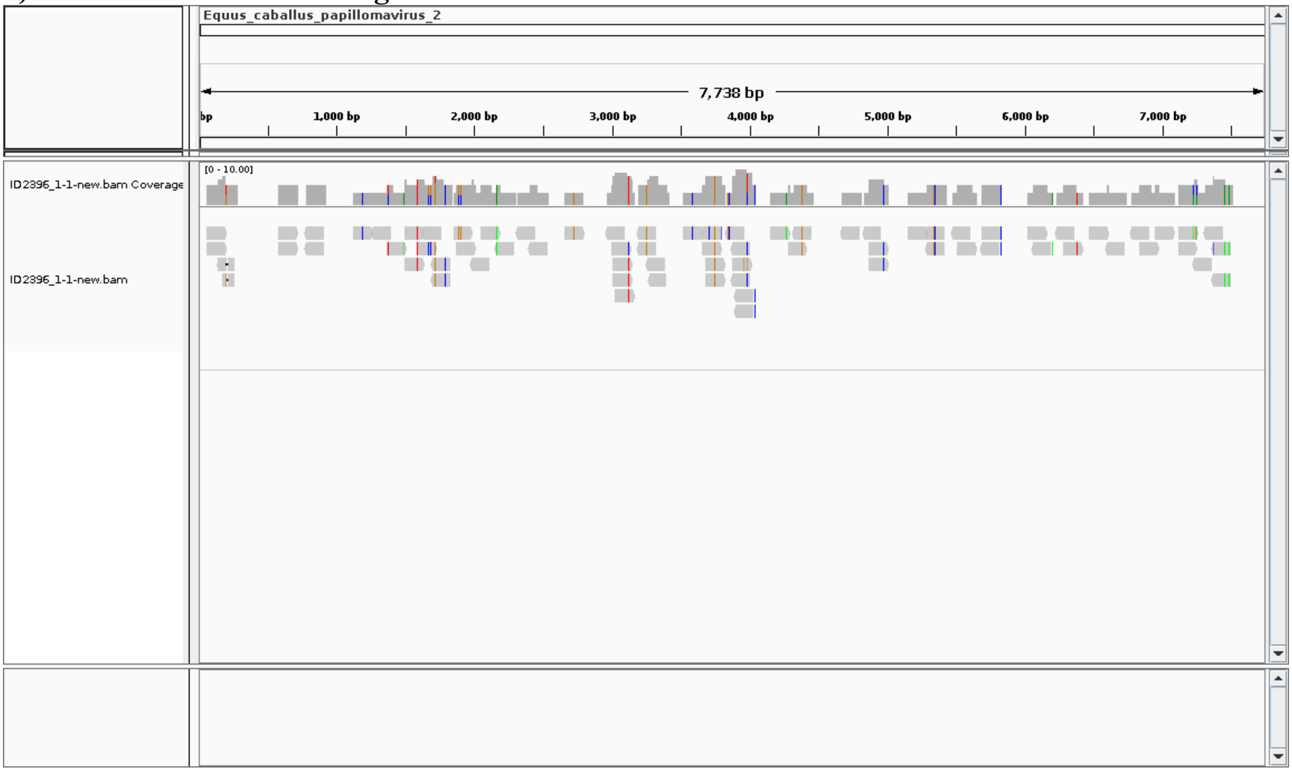

b) ID2396 4-4 raw reads aligned to the consensus reconstructed sequence

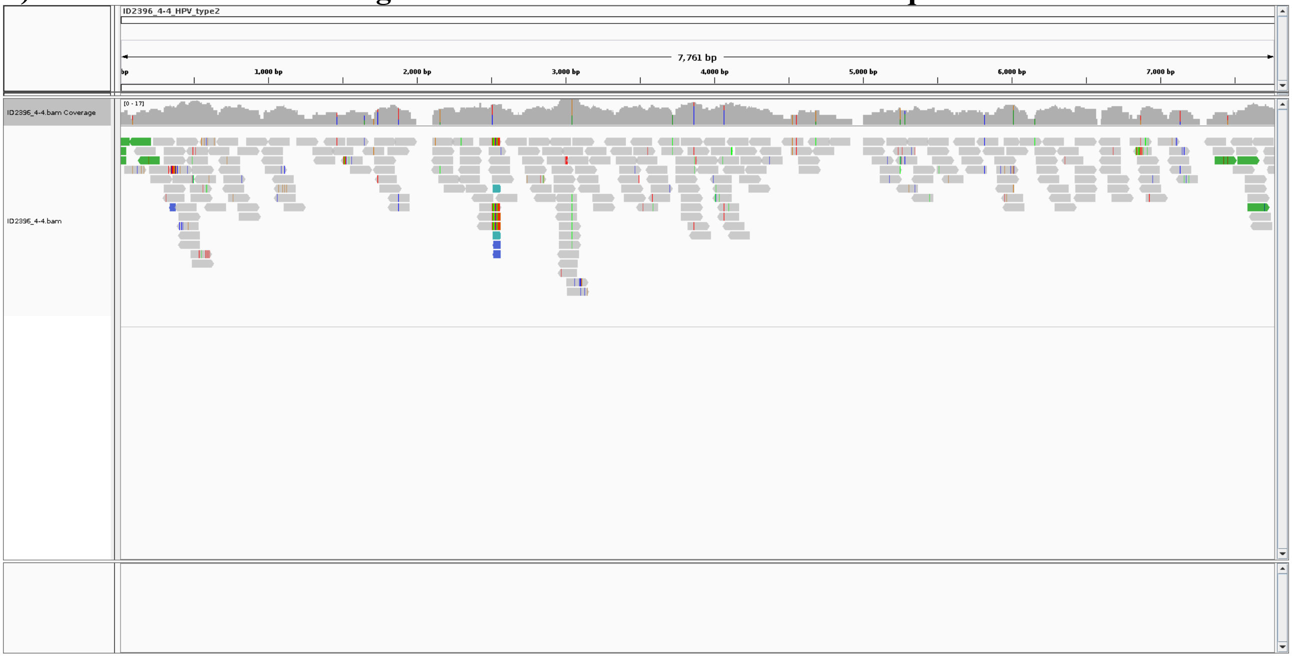

c) ID2396 7-7 raw reads aligned to the reconstructed EcPV2 sequence

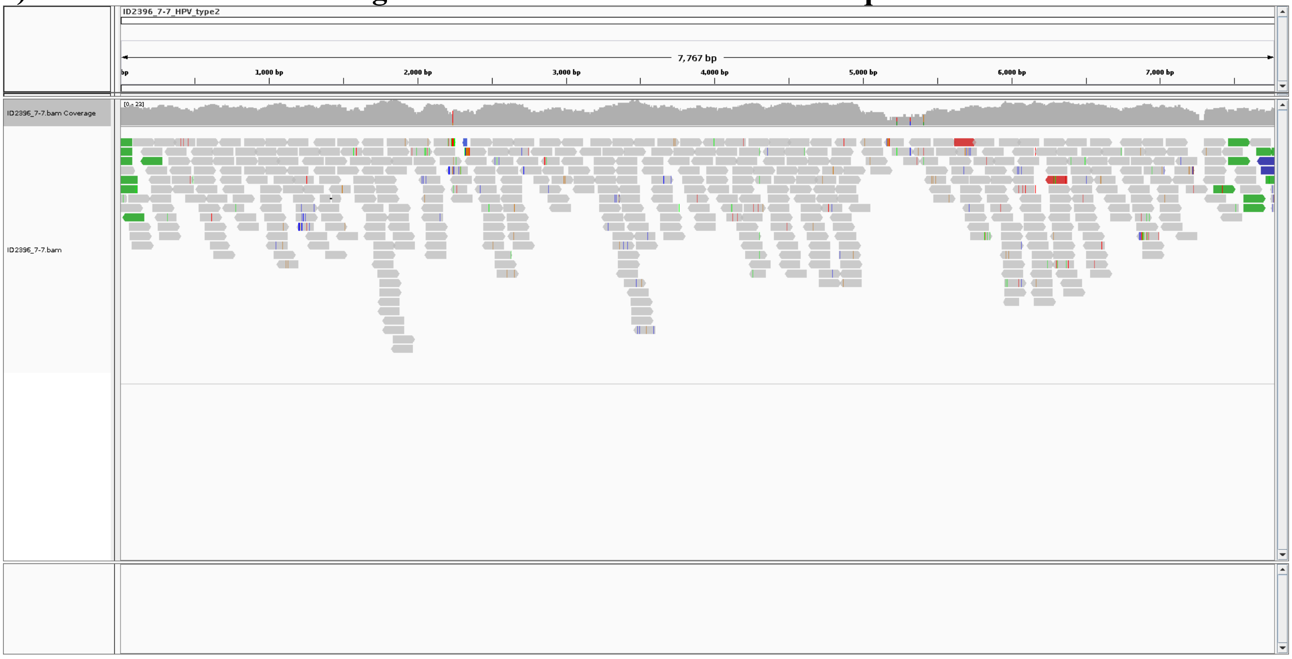

e) ID2396 10-10 raw reads aligned to the reconstructed EcPV2 sequence

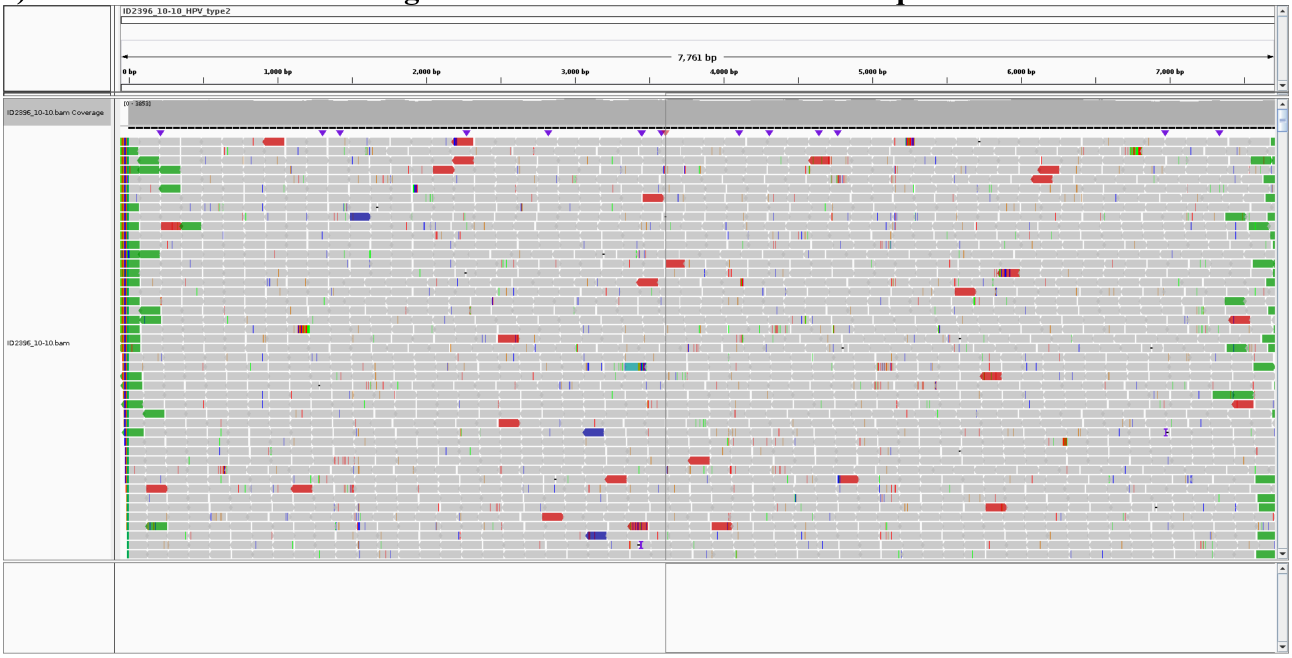

d) ID2396 12-12 raw reads aligned to the reconstructed EcPV2 sequence

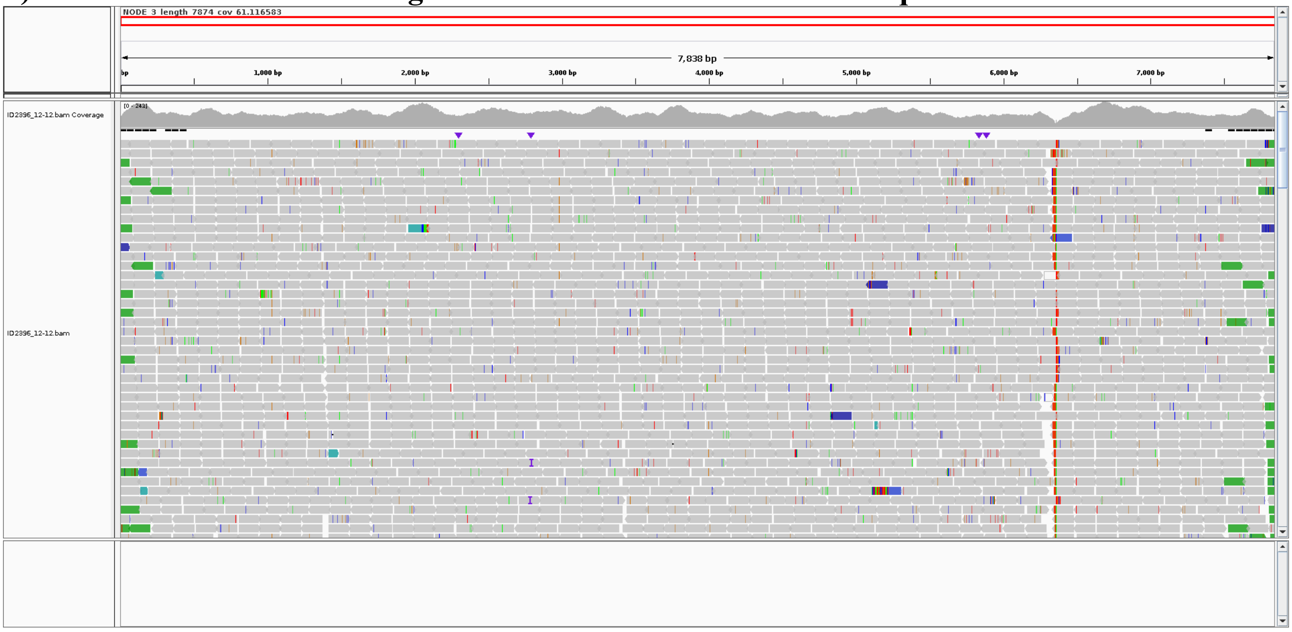

Supplement: Supplementary file 1 [file viruses-14-01696-s001.zip › FigureS3_revised.pdf]
