# Supplementary material for: Detection of Equus Caballus Papillomavirus Type-2 in Asymptomatic Italian Horses
Source: Viruses. 2022 Jul 31;14(8):1696. doi: 10.3390/v14081696 (PMC9412442; doi:10.3390/v14081696)
Supplement: Supplementary file 1 [file viruses-14-01696-s001.zip › Supplementary_revised.pdf]

Figure S1: Animal breed consistencies

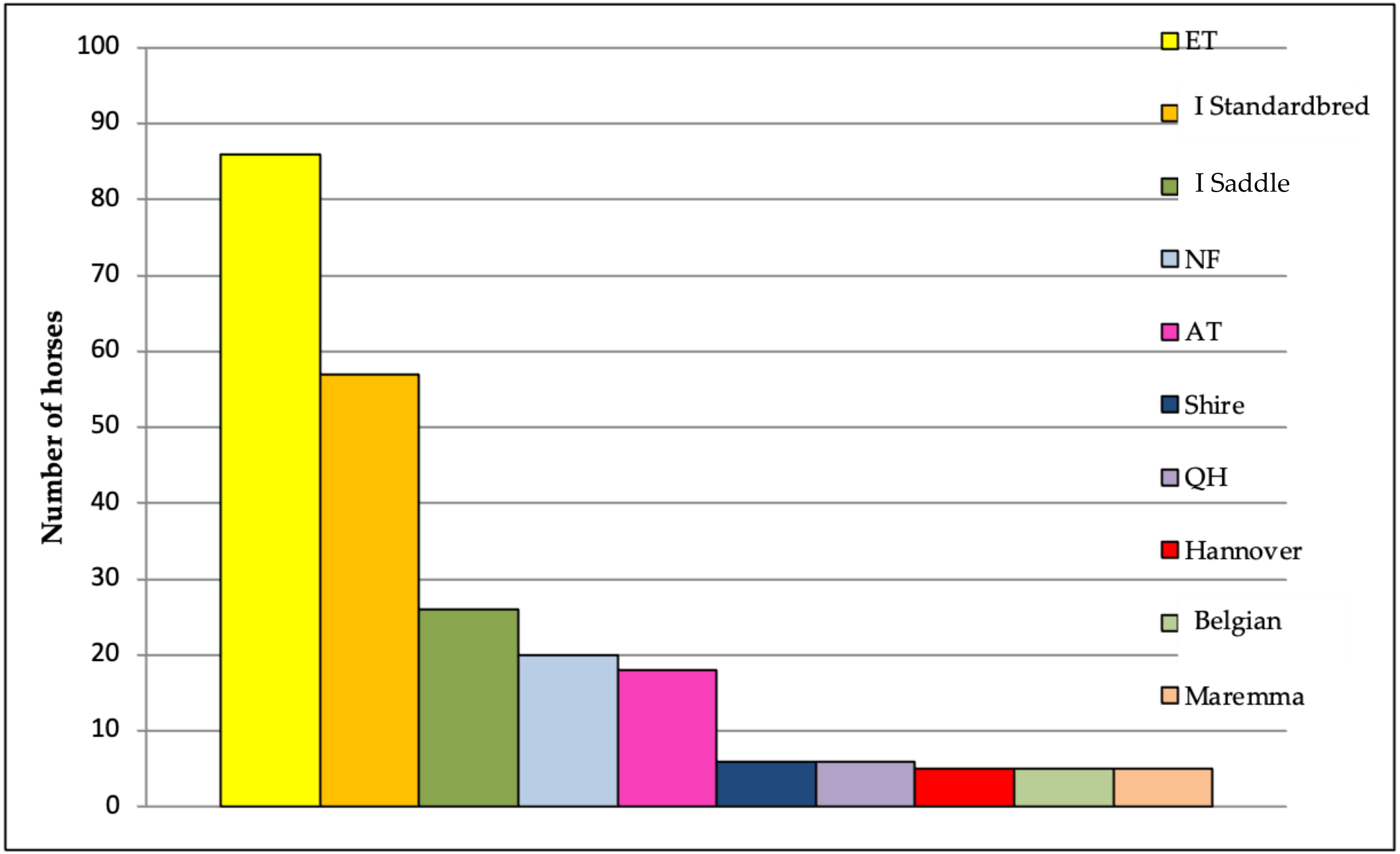

NF: Data not found

Figure S2: Animal origin

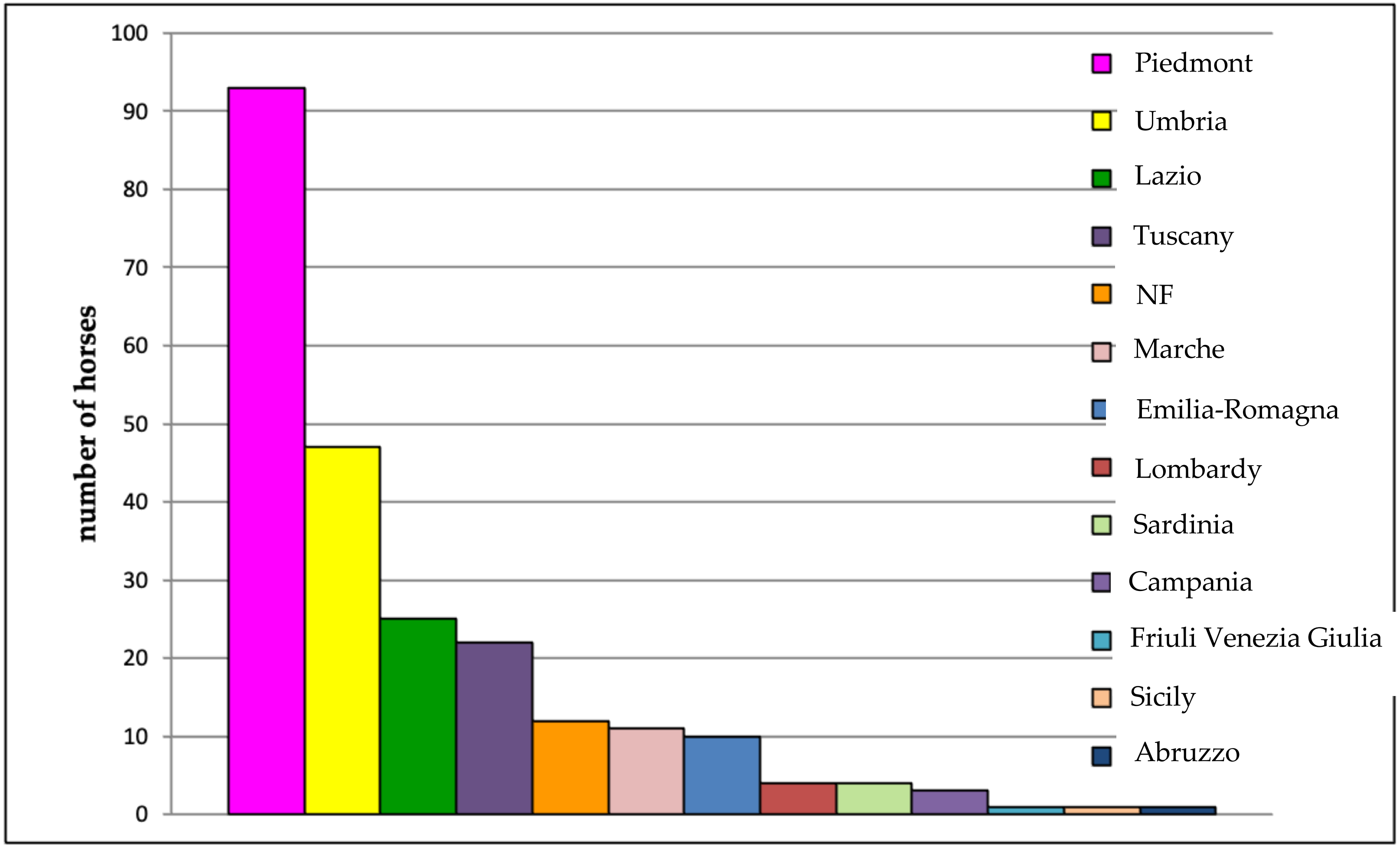

NF: Data not found

Table S1: List of the EcPV isolates and their related accession numbers included in the in house database used for the NGS data analysis

EcPV type	Accession number
Equus caballus papillomavirus type 1	AF498323.1
Equus caballus papillomavirus type 1	NC_003748.1
Equus caballus papillomavirus type 1 strain 150904	MF288893.1
Equus caballus papillomavirus type 1 isolate G2	MN164462.1
Equine papillomavirus 2	EU503122.1
Equine papillomavirus 2	NC_012123.1
Equine papillomavirus 2 isolate Zurich 2009	HM461973.1
Equus caballus papillomavirus 2 isolate XJ-ks1391	MW410986.1
Equine papillomavirus 3	NC_017862.1
Equine papillomavirus 3 isolate Haflinger	GU384895.1
Equus ferus caballus papillomavirus type 4	NC_020085.1
Equus ferus caballus papillomavirus type 4	JQ031032.1
Equus ferus caballus papillomavirus type 5	JQ031033.1
Equus ferus caballus papillomavirus type 5	NC_020084.1
Equine papillomavirus type 6	JQ965698.1
Equine papillomavirus type 6	NC_020500.1
Equus ferus caballus papillomavirus type 7	JX035935.1
Equus ferus caballus papillomavirus type 7	NC_020501.1
Equus caballus papillomavirus type 8 strain NCSU	KU963288.1
Equus caballus papillomavirus type 8 strain NCSU	NC_031756.1
Equus caballus papillomavirus 9 strain SW	MN117918.1

Table S2: Animals positive for the detection of EcPV2-L1 and subsequently analysed for the oncogene EcPV2-E2, EcPV2-E6 and EcPV2-E7 by qPCR

L1	E2	E6	E7
34.6±0.7	ND	32.5±0.3	32.8±0.4
32.4±0.4	33.8±0.5	30.7±0.3	30.9±0.5
35.1±0.8	37.4±0.7	33.3±0.7	34.2±0.5
35.6±0.2	ND	34.9±1.0	34.2±0.8
37.9±0.1	36.3±0.8	35.6±0.6	35.1±0.5
37.3±0.4	ND	34±0.2	34±0.4
32.4±1	36.6±0.4	33.1±0.5	32.8±0.4
16.5±0.7	19.3±0.4	16.5±0.2	17.5±0.3
28.4±0.3	34.9±1.3	31.7±1.7	28.7±0.6
32.6±0.2	ND	34.9±1.2	32.9±1.8
34.5±0.5	30.6±0.2	27.8±0.1	28.6±0.1
34.31±0.3	37.3±0.8	35.1±0.7	35.5±0.6
30.8±1.4	35.9±1.7	31.7±1.8	32.1±1.1
34.7±0.2	36.8±0.7	34.2±0.5	34.9±0.1
36.0±0.9	ND	37.6±0.7	37.1±0.9
32.1±0.2	ND	32.0±0.4	32.0±0.1
34.5±0.2	35.2±0.7	32.2±0.7	33.7±1.1
37.2±0.6	38±0.6	35.4±0.5	36±0.9
38.1±0.8	ND	37.4±0.4	38±0.6
32.5±0.8	38.0±0.6	34.1±0.2	33.8±0.3
29.3±0.8	ND	28.8±0.3	28,.±0.3
35.6±0.3	35.9±0.8	34.8±0.3	34.9±0.3
33.6±0.2	37.6±1.1	34.01±0.3	33.7±0.6
27.6±0.4	30.8±0.5	27.5±0.2	27.4±0.3

L1	E2	E6	E7
37.0±1	ND	36.5±0.8	36.2±0.7
29.6±0.3	38.2±0.5	36.5±0.4	36.4±0.4
29.5±0.4	32.0±0.3	29.0±0.2	29.9±0.2
29.3±0.8	38.4±0	28.8±0.3	28.8±0.3
23.8±1.0	27.1±0.2	24.2±0.3	24.9±0.3
22.5±0.3	25.1±1.0	22.3±0.5	22.1±0.5
35.9±1.0	38±0.8	35.8±1.2	35.2±1.3
26.3±0.3	28.2±0.9	25.7±0.6	25.5±0.4
36.3±0.7	ND	35.4±0.6	34.7±0.4
32.4±0.2	37.1±1.7	32.1±0.0	32.1±0.4
32.1±0.2	35.0±0.8	32.0±0.3	32.3±0.1
37.1±0.8	ND	36.0±0.9	36.7±1.4
34.3±1.2	35.5±0.2	33.0±0.6	33.2±1.1
33.1±0.1	36.6±1.2	33.1±0.6	32.8±0.5
22.9±1.4	30.5±1.0	23.3±0.5	23.6±0.3
31.4±1.6	34.9±1.7	31.8±0.6	31.7±0.7
36.9±1	ND	37.3±1.2	37.2±1.7
30.5±0.6	32.5±0.7	30±0.1	29.8±0.3
32.1±1.1	35.4±1.6	32.5±0.7	32.2±0.6
35.4±0.7	38.3±0.5	35.8±1.1	34.6±0.8
30.6±1.7	33.8±1.7	30.9±0.5	30.9±0.5
26.9±1.5	ND	27.3±0.4	27.4±0.5
33.1±0.4	ND	34±0.3	33.8±0.4
29.5±0.1	31.6±0.1	29.2±0.1	28.6±0.2

L1	E2	E6	E7
28.8±0.2	31.5±1.1	28.8±0.4	28.4±0.5
28.1±0.3	30.7±1.1	28.2±0.6	27.9±1.1
30.8±0.3	33.0±0	30.5±0.1	30.3±0.2
32.7±1.2	36.2±1.3	31.2±1.7	36.1±1.1
30.7±0.4	32.4±0.9	29.8±0.2	29.6±0.1
31.5±0.3	33.4±0.7	31.1±0.7	30.4±0.2
33.6±0.6	ND	33.1±0.2	32.6±0.1
35±0.3	36.8±0.5	34.6±0.9	34±0.5
31.5±0.1	33.9±0.4	30.8±0.5	30.6±0.3
28.9±0.3	31.3±0.8	28.6±0.3	28.3±0.1
34±1.2	36±0.9	33.1±0.2	32.6±0.4
35.3±0.6	ND	34.7±0.2	34.±0.3
36.3±0.9	ND	35.3±0.1	35.2±0.9
23.9±0.5	25.7±0.1	23.8±0.2	23.5±0.1
16.5±0.5	18.7±0.3	17±1.2	15.9±0.3
31±0.5	32.7±0.2	30.5±0.5	30.1±0.4
34±0.1	36±0.8	33.8±0.4	33.2±0.9
28.8±0.3	31±0.1	28.9±0.2	28.8±0.1
32.3±0.4	34.6±0.8	32.1±0.1	31.3±0.4
26.6±0.5	29.1±0.1	26.6±0.1	26.2±0.1
30.8±1.7	32.7±1.5	30.3±1.6	29.8±1.7
31.2±0.5	33.5±0.1	31±0.1	30.6±0.1
32.1±0.3	34.6±0.1	31.9±0.2	26.4±0.2

ND: Data not determined

Table S3: Sanger data analysis summary

Sample name	BLAST	coverage	Identity	p value
56	Equuscaballus papillomavirus 2	100%	99.26%-99,45%	0.0
66	Equuscaballus papillomavirus 2	100%	99.27%-99,45%	0.0
71	Equuscaballus papillomavirus 2	99-100 %	99.82%	0.0
82	Equuscaballus papillomavirus 2	97-99%	99.64%-99,82%	0.0
125	Equuscaballus papillomavirus 2	97-100%	99.64%-99,82%	0.0
165	Equuscaballus papillomavirus 2	96-100%	99.65%-99,82%	0.0
170	Equuscaballus papillomavirus 2	99-100%	99.27%-99,64%	0.0
171	Equuscaballus papillomavirus 2	99-100%	99.07%-99,47%	0.0
166	Equuscaballus papillomavirus 2	97-100%	99.64%-99,82%	0.0

Table S4: NGS data analysis summary. For each samples are indicated the geographic origins, the raw reads before and after filtering, as well as the coverage on the Reference EcPV2 geome (for ID2396_1-1) or the newly reconstructed sequences.

Sample Name	Geographic origin	Total raw reads	Total trimmed reads	Total filtered reads	Breadth coverage	Depth coverage
80057_ID2396_1-1	Roma, Lazio	91.851.832	74.210.728	1.138.480	70.46%	1.31x
80060_ID2396_4-4	Vigone, Piemonte	24.508.308	17.447.038	183.722	93.36%	4,66x
80063_ID2396_7-7	Biella, Piemonte	36.642.818	27.546.452	1.383.726	100%	8,91x
80066_ID2396_10-10	Perugia, Umbria	66.724.240	47.236.522	1.317.748	100%	2289.62x
80068_ID2396_12-12	Emilia Romagna	20.235.840	13.617.152	433.644	100%	139x

Table S5: Comparison between some selected samples positive for DNA expression of EcPV2-L1 and oncogenes (E2, E6 and E7) by qPCR and the gene expression of EcPV2-L1 and E6 by RTqPCR

DNA				cDNA	
**L1**	**E2**	**E6**	**E7**	**L1**	**E6**
16.4±0.2	19.0±0.2	16.4±0.1	17.3±0.1	27.0±0.1	31.0±0.7
28.9±0.3	31.3±0.8	28.6±0.3	28.3±0.1	35.5±0.9	ND
29.5±0.4	32.0±0.3	29.0±0.2	29.9±0.2	35.5±0.7	ND
29.5±0.1	31.6±0.1	29.2±0.1	28.6±0.2	35.4±0.5	ND
30.5±0.6	32.5±0.7	30.0±0.1	29.8±0.3	35.7±0.9	ND
30.7±0.4	32.4±0.9	29.8±0.2	29.6±0.1	39.0±2.5	ND
31.0±0.5	32.7±0.2	30.5±0.5	30.1±0.4	39.7±1.1	ND
31.5±0.1	33.9±0.4	30.8±0.5	30.6±0.3	ND	ND
31.5±0.3	33.4±0.7	31.1±0.7	30.4±0.2	36.4±0.6	ND
32.1±0.2	ND	32.0±0.4	32.0±0.1	37.1±0.8	ND
32.1±0.2	35.0±0.8	32.0±0.3	32.3±0.1	ND	ND
32.3±0.4	34.6±0.8	32.1±0.1	31.3±0.4	37.6±0.3	ND
33.1±0.1	36.6±1.2	33.1±0.6	32.8±0.5	36.9±0.7	ND
33.6±0.2	37.6±1.1	34.01±0.3	33.7±0.6	ND	ND
33.6±0.6	ND	33.1±0.2	32.6±0.1	ND	ND
34.0±1.2	36.0±0.9	33.1±0.2	32.6±0.4	ND	ND
34.0±0.1	36.0±0.8	33.8±0.4	33.2±0.9	ND	ND
34.3±1.2	35.5±0.2	33.0±0.6	33.2±1.1	ND	ND
34.5±0.5	306±0.2	27.8±0.1	28.6±0.1	35.0±0.5	ND
34.5±0.2	35.2±0.7	32.2±0.7	33.7±1.1	ND	ND
34.7±0.2	36.8±0.7	34.2±0.5	34.9±0.1	35.5±0.5	ND
35.0±0.3	36.8±0.5	34.6±0.9	34.0±0.5	ND	ND
35.4±0.7	38.3±2.5	35.8±1.1	34.6±0.8	ND	ND
36.3±0.9	ND	35.3±0.1	35.2±0.9	ND	ND
37.2±1.2	ND	37.3±1.2	37.2±2.1	ND	ND

L1 positive for DNA and cDNA

L1 positive only for DNA

ND: Data not determined
